# Method to Reduce the False-Positive Rate of Loss of Resistance in the Cervical Epidural Region

**DOI:** 10.1155/2016/9894054

**Published:** 2016-03-29

**Authors:** Young Uk Kim, Doohwan Kim, Jun Young Park, Jae-Hyung Choi, Ji Hyun Kim, Heon-Yong Bae, Eun-Young Joo, Jeong Hun Suh

**Affiliations:** ^1^Department of Anesthesiology and Pain Medicine, Catholic Kwandong University of Korea College of Medicine, International St. Mary's Hospital, Incheon, Republic of Korea; ^2^Department of Anesthesiology and Pain Medicine, Asan Medical Center, University of Ulsan College of Medicine, 88 Olympic-ro 43 Gil, Songpa-gu, Seoul 05505, Republic of Korea

## Abstract

*Background*. The cervical epidural space can be detected by the loss of resistance (LOR) technique which is commonly performed using air. However, this technique using air has been associated with a high false-positive LOR rate during cervical interlaminar epidural steroid injections (CIESIs).* Objective*. We investigated whether the detection of LOR with contrast medium might reduce the false-positive LOR rate on the first attempt.* Methods*. We obtained data retrospectively. A total of 79 patients were divided into two groups according to the LOR technique. Groups 1 and 2 patients underwent CIESI with the LOR technique using air or contrast medium. During the procedure, the injection technique (median or paramedian approach), final depth, LOR technique (air or contrast), total number of LOR attempts, and any side effects were recorded.* Results*. The mean values for the total number of LOR attempts were 1.38 ± 0.65 (Group 1) and 1.07 ± 0.25 (Group 2). The false-positive rate on the first attempt was 29.4% and 6.6% in Groups 1 and 2, respectively (*P* = 0.012).* Conclusions*. The use of contrast medium for LOR technique is associated with a lower rate of false-positivity compared with the use of air.

## 1. Introduction

Cervical interlaminar epidural steroid injections (CIESIs) are widely used to treat chronic axial neck and radicular pain from spinal stenosis, herniated discs, and acute pain conditions that involve the upper extremities, posterior neck, and head [1–5]. Loss of resistance (LOR) is the most commonly used technique for identifying the epidural space. It relies on sudden LOR to the injection of either air or liquid. To improve the accuracy of needle placement, many clinicians prefer a fluoroscopic-guided LOR technique [4]. However, several studies of CIESI have reported a 30–53% false-positive rate with air on the first attempt because of high rate of variability and discontinuity in the ligamentum flavum [6], which plays an essential role in the cervical region [1, 4, 7, 8]. Although CIESI is considered to be a relatively safe procedure, the high rate of false-positivity for LOR in the cervical region is associated with significant complications [9].

The primary aim of this study was to examine whether the use of air or contrast medium for the LOR technique could diminish the false-positive rate on the first attempt. The false-positive rate represents a loss prior to actually entering the epidural space in this study. We predicted that LOR detection with contrast medium would increase the accuracy of CIESI because of the high viscosity and surface tension of this medium.

## 2. Methods

The research protocol was approved by our institutional review board and registered at the University of Ulsan, Seoul, Republic of Korea, with the assigned number S2014-2040-0001. Procedure records for all relevant patients at Asan Medical Center were requested between June 2014 and June 2015. We retrospectively reviewed these patients medical records, including demographic data (sex, age, weight, and height) and chief complaints (pain, paresthesia, or both pain and paresthesia with weakness). Short abstracts were written based on these records, including details of the procedure and technique. In our Department of Pain Medicine, cervical spine interventions are recorded using a consistent itemized format. During each procedure, the injection technique (median or paramedian approach), final depth, the LOR technique (air or contrast), total number of LOR procedures, and any side effects, such as a dural puncture, were recorded.

We divided subjects into two groups according to the LOR technique that was used; Group 1 patients underwent CIESI with the LOR technique using air, while Group 2 patients underwent CIESI with the LOR technique using a contrast agent (Omnipaque 300 [iohexol, 300 mg iodine per mL]; GE Healthcare, Little Chalfont, UK). Inclusion was limited to patients with a history of chronic functional limitations of the upper extremity, shoulder, and posterior neck because of pain that was of at least 3-month duration and resulted from cervical disc herniation or uncovertebral joint narrowing. In addition, only patients 20 years or older were included. All patients with prior cervical surgery, dislocations, and fractures of the cervical spine were excluded. Fluoroscopically guided CIESI was performed by one pain specialist who had completed more than 1000 ESI procedures at Asan Medical Center in a sterile operating room. All patients were positioned in a prone position with the neck flexed, facedown, with two gel-pads stacked under the chest.

**Figure 1:**
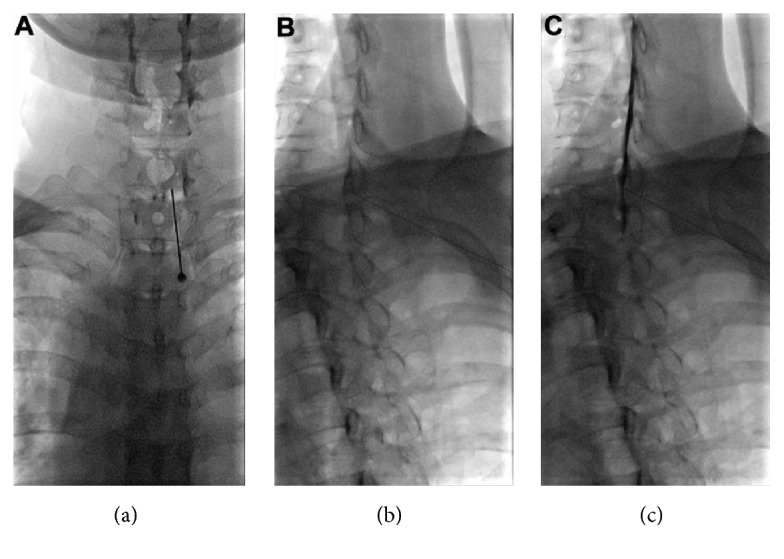
AP and the contralateral oblique views under fluoroscopy. AP view (a) and the contralateral oblique view at 50 degrees ((b) and (c)) under fluoroscopy; the contralateral laminae are seen in complete cross-section view ((b) and (c)). The needle can be seen transversing between the inferior and superior laminae with the needle tip directed toward the spinolaminar line (b). And epidural space was confirmed with contrast medium injection (c).

In Group 1, the 22 G Tuohy needle (Hakko; Chikuma-shi, Nagano-ken, Japan) was connected to a syringe that contained 2.5 mL air, while in Group 2, the 22 G Tuohy needle was filled with contrast medium (0.2 mL contrast) and connected to a syringe that contained 2.5 mL air. Under fluoroscopic visualization, the interlaminar space between C7 and T1 was identified. After anesthetizing the skin with 1% lidocaine, a 22-gauge Tuohy needle was inserted via a midline or paramedian approach that matched the symptomatic side at the C7-T1 interspace (Figure 1(a)).

Cervical contralateral oblique view was used during the procedure. To obtain optimal contralateral oblique view, fluoroscopy was rotated in AP/lateral plane until a parallel view of the contralateral lamina was achieved. When X-ray beam was parallel to the ventral margin of the lamina, we can better visualize the spinolaminar line. The needle was slowly and carefully advanced through the ligamentum flavum using the LOR technique after the level is confirmed. In this fluoroscopic vision, the needle can be seen transversing between the inferior and superior laminae with the needle tip directed toward the spinolaminar line (Figure 1(b)). The posterior epidural space was entered between C7 and T1. Confirmation of correct epidural placement occurred when the contrast agent (Omnipaque 300, GE Healthcare, Little Chalfont, UK) was seen to spread evenly throughout the epidural space (Figure 1(c)). A mixture of 5 mg dexamethasone and 3–6 mL 0.125% bupivacaine was then injected into the epidural space.

### 2.1. Statistical Analysis

Data were expressed as means ± standard deviation (SD). We used an unpaired* t*-test to compare the means of the two study groups. Comparisons of the success rate were made using the Pearson chi-square test and Fisher's exact test. *P* values <0.05 were considered to indicate statistically significant differences. SPSS for Windows version 21 (IBM SPSS Inc., Chicago, IL) was used for statistical analysis.

## 3. Results

A total of 79 patients were included in our current analyses. The demographics of the study subjects managed with LOR using air or contrast are listed in Table 1. There were no significant differences in any of the patient characteristics between the two study groups (Table 1). The total number of LOR attempts is indicated in Table 2. The mean values for the total number of LOR attempts were 1.38 ± 0.65 (air group) and 1.07 ± 0.25 (contrast group). In Group 1, 24 patients exhibited successful results (successful identification of the epidural space by the LOR technique on the first attempt) and 10 patients were classified as a fail (success rate 70.6% and false-positive rate 29.4%). In contrast, in Group 2 there were 42 successes and 3 fails (success rate 93.4% and false-positive rate 6.6%; Table 3). This difference was significant (*P* = 0.012, Fisher's exact test). The success rate was significantly lower in the air group (Group 1) than in the contrast group (Group 2; odds ratio = 0.171; 95% confidence interval [CI], 0.043 to 0.684).

## 4. Discussion

Although CILESIs are commonly used procedures in the diagnosis and treatment of painful disorders of the cervical spine [10], they have been associated with complications, such as nonpositional headache, facial flushing, vasovagal episodes, and increased axial neck pain. Additionally, major complications, such as epidural hematoma, subdural hematoma, permanent spinal cord injury, and death, can occur during the procedure [11, 12]. To improve patient safety, many clinicians recommend performing the procedure under fluoroscopic guidance [4, 13–15]. Fluoroscopic-guided procedures allow the injection of contrast medium to verify the correct spread of the injectate in the cervical epidural space and to exclude intravascular or intrathecal injection [16]. However, fluoroscopic guidance CILESI does not diminish the false-positive rate on the first attempt. No previous prospective or retrospective studies have compared the false-positive rate in the cervical region when air or contrast is used.

Stojanovic et al. reported a 30–53% false-positive LOR rate during the first attempt to identify the epidural space [4]; these authors performed the LOR technique using air. Our current findings are consistent with that previous report and indicate a false-positive rate of 29.4% on the first attempt to identify the epidural space by LOR detection using air. We found in comparison that a contrast-filled Tuohy needle for the LOR technique was associated with a lower false-positive rate (6.6%). We used an iohexol preparation (Omnipaque 300, GE Healthcare, Little Chalfont, UK), which contains 647 mg iohexol (equivalent to 300 mg organic iodine per mL) and is provided as a colorless to pale-yellow, pyrogen-free sterile solution. The osmolarity of iohexol is 465 mOsm/L—approximately 1.5-fold greater than that of normal saline. A cadaveric study has demonstrated that liquid injected under pressure at the moment of LOR can potentially thrust the dura away from the needle tip, thereby helping to avoid dural puncture when compared to air [17]. We speculate that contrast is more effective than saline in this regard because of its greater osmolality and viscosity (the Omnipaque 300 viscosity at 37.0°C is 6.3 centipoise (cp), whereas the normal saline viscosity at 37.0°C is 0.8 cp).

We did not compare contrast with saline because Segal and Arendt have reported that the isotonic saline returning from a blocked needle might sometimes be mistaken for cerebrospinal fluid when the Tuohy needle is not yet in the epidural space, resulting in a failed block [3]. In the cervical region, anatomical studies have shown not only high rate of discontinuity, but also anatomic variability in the ligamentum flavum, which plays an inevitable role in the LOR technique. This morphological variability is associated with a higher false-positive rate during CIESI procedures [7, 18, 19].

According to the epidural steroid injections safety recommendations, entry at C7-T1 level is recommended, but preferably not higher than the C6-C7 level because of the variability and discontinuity of the ligamentum flavum. Therefore, we performed CILESI at C7-T1 level following recommendations [20, 21]. Although fluoroscopic-guided techniques increase the procedure precision and help to confirm correct needle placement [7, 14, 22], the LOR technique with air has a high rate of false-positive LOR on the first attempt.

It is known that performing the LOR technique with air can increase the incidence of complications, such as venous air emboli, nerve root compression, and subcutaneous emphysema [23, 24]. Epidural air can migrate around the nerve roots. Additionally, depending on its location, neurological complications, such as cervical root compression, multiradicular syndrome, and even paraplegia, can occur [12]. Therefore, LOR with air injection can cause radiculopathy. The potential complications of air also include pneumocephalus and headaches in patients who receive epidural anesthesia. These headaches from pneumocephalus result from an accidental dural puncture during epidural placement using air for the LOR technique [25]. To prevent these complications, the LOR technique using contrast medium should be considered, which could potentially diminish the incidence of certain air-related complications. Furthermore, the cervical epidural space can be detected directly by contrast medium that is distributed using a Tuohy needle without additional contrast agent and could thus help to reduce radiation exposure. Finally, we detected a statistically significant reduction in the number of attempts required to locate the epidural space with LOR using contrast medium instead of air.

We used contralateral oblique view image because the shoulders can obscure visual identification of epidural space in true lateral view image of C7-T1 [26]. Gill et al. have demonstrated that the contralateral oblique view in the cervicothoracic level is superior to the lateral view for the aim of needle tip visualization and in providing a consistent landmark for evaluating the epidural space [27].

Our study had several limitations of note. First, it was not a prospective, controlled study. However, follow-up records were recorded on an itemized medical chart such that the follow-up data could be more systemically gathered in a manner similar to that used in prospective studies. Second, the sample size was relatively small. Future studies should include a larger number of subjects to further evaluate differences between air-only, saline-only, and contrast-only LOR techniques. The findings of our present pilot study may yield insights into the expected differences in these LOR methods and help to enhance the statistical power of future studies. Third, the false-positive rate only represents a loss prior to actually entering the posterior epidural space in our study. This false-positive does not include intrathecal or intramedullary access.

## 5. Conclusions

The use of contrast medium for the LOR technique is associated with a lower false-positive rate on the first attempt compared with air. Additionally, LOR techniques using contrast can shorten the procedure time and limit radiation exposure due to the reduced false-positive rate. We thus strongly recommend this approach when performing CIESI.

## Figures and Tables

**Table 1:** Study patient characteristics and the final depth of the cervical epidural space.

Parameter	Air group (*N* = 34)	Contrast group (*N* = 45)	*P* value
Age (yr)	55.65 ± 12.52	52.58 ± 13.87	0.441
Height (cm)	167.85 ± 8.03	165.68 ± 7.35	0.361
Weight (kg)	65.81 ± 8.70	62.84 ± 10.01	0.295
BMI (kg/m^2^)	21.71 ± 6.77	22.79 ± 2.49	0.509
Diagnosis (*N*)			
C-HIVD	22	34	
C-SS	12	11	
Needle tip approach (*N*)			
Median approach	0	1	
Paramedian approach	34	44	
Right	18	24	
Left	16	20	
Final depth (cm)	6.25 ± 0.72	6.69 ± 0.85	0.062

BMI: body mass index; C-HIVD: cervical herniated intervertebral disc; C-SS: cervical spinal stenosis.

**Table 2:** Total number of LOR procedures and unintentional dural punctures.

Variable	Air group (*N* = 34)	Contrast group (*N* = 45)
Total number of LOR		
1	24	42
2	7	3
3	3	0
4 or more	0	0
Mean values	1.38 ± 0.65	1.07 ± 0.25
Unintentional dural puncture (*N*)	0	0

LOR: loss of resistance.

**Table 3:** Comparison of the success rate on the first attempt.

	Group		Total
	Air	Contrast	
First attempt			
Success	24	42	66
Failure	10	3	13
Total	34	45	79

## Additional Points

We surveyed total number of loss of resistance attempts in patients who underwent cervical interlaminar epidural steroid injection with the loss of resistance technique using either air (Group 1) or contrast medium (Group 2). The loss of resistance technique using contrast medium showed a lower false-positive rate compared with the use of air. This technique can reduce the procedure time and limit radiation exposure on account of the reduced false-positive rate. In consequence, loss of resistance maneuver using contrast medium should be considered when performing cervical interlaminar epidural steroid injection.
